# Spectral Decompositions of Multiple Time Series: A Bayesian Non-parametric Approach

**DOI:** 10.1007/s11336-013-9354-0

**Published:** 2013-10-24

**Authors:** Christian Macaro, Raquel Prado

**Affiliations:** 1 SAS Institute, Cary, NC USA; 2 Department of Applied Mathematics and Statistics, University of California, Santa Cruz, Santa Cruz, USA

**Keywords:** spectral one-way and two-way models, Bayesian nonparametrics, Whittle’s approximation, Bernstein–Dirichlet priors

## Abstract

We consider spectral decompositions of multiple time series that arise in studies where the interest lies in assessing the influence of two or more factors. We write the spectral density of each time series as a sum of the spectral densities associated to the different levels of the factors. We then use Whittle’s approximation to the likelihood function and follow a Bayesian non-parametric approach to obtain posterior inference on the spectral densities based on Bernstein–Dirichlet prior distributions. The prior is strategically important as it carries identifiability conditions for the models and allows us to quantify our degree of confidence in such conditions. A Markov chain Monte Carlo (MCMC) algorithm for posterior inference within this class of frequency-domain models is presented.

We illustrate the approach by analyzing simulated and real data via spectral one-way and two-way models. In particular, we present an analysis of functional magnetic resonance imaging (fMRI) brain responses measured in individuals who participated in a designed experiment to study pain perception in humans.

## Introduction

Time series data from several subjects that can be classified into groups according to a set of features are oftentimes recorded in clinical and non-clinical studies. For instance, a set of brain signals recorded from several individuals may be grouped by the type of stimulus each individual received in a given experimental setting. In such cases, one of the main goals of the time series analysis is to determine if there are differences in the spectral characteristics of the signals across the groups.

More specifically, suppose that a collection of time series $\{ y_{i_{1},i_{2},h}(t)\}$ are recorded during a particular experimental setting in which *i*_1_ indexes the level of some factor, say A, *i*_2_ indexes the level of some other factor, say B, and *h* indexes the time series within given levels of the A and B factors, where *i*_1_=1:*N*_1_, *i*_2_=1:*N*_2_, $h=1:H_{i_{1},i_{2}}$, and *t*=1:*T*. Such data may arise in designed experiments where factor A is a given treatment or experimental condition—e.g., the type of stimulus in a neuroscience experiment or the disease level—, factor B is another treatment or experimental condition, and *h* indexes the individuals who were assigned to levels *i*_1_ and *i*_2_ of the A and B factors, respectively. We are interested in fitting models that allow us to characterize the spectral features of the time series in terms of effects associated to the levels of the factors and, possibly also, in terms of effects associated with the interaction of such factors. Assume for instance that each time series can be decomposed as

1 $$ y_{i_1,i_2,h}(t) = \beta_{i_1}^{(1)}(t) + \beta^{(2)}_{i_2}(t) + \varepsilon_{i_1,i_2,h}(t), $$

where the unobserved processes $\beta_{i_{1}}^{(1)}(t)$ and $\beta_{i_{2}}^{(2)}(t)$ are assumed to be uncorrelated, i.e., $\mbox{Cov}(\beta_{i_{1}}^{(1)}(t),\beta_{i_{2}}^{(2)}(t))=0$ for all *i*_1_,*i*_2_ and all *t*=1:*T*. Furthermore, for a given *k* with *k*=1,2 assume that $\mbox{Cov}(\beta_{i}^{(k)}(t),\beta_{j}^{(k)}(t))=0$ when *i*≠*j* for all *t*. The $\varepsilon_{i_{1},i_{2},h}(t)$s are idiosyncratic—i.e., individual specific—error series assumed to be independent of the other unobserved processes and also mutually independent. This implies that the spectral density of $y_{i_{1},i_{2},h}(t)$, which is the Fourier transform of the theoretical autocovariance function of the $y_{i_{1},i_{2},h}(t)$ process and is denoted as $f_{y_{i_{1},i_{2},h}}^{*}(\lambda)$, can be written as

2 $$ f_{y_{i_1,i_2,h}}^*(\lambda) = f_{i_1}^{*,(1)}(\lambda) + f_{i_2}^{*,(2)}(\lambda) + f^*_{\varepsilon_{i_1,i_2,h}}(\lambda), $$

where $f_{i_{1}}^{*,(1)}(\lambda)$ and $f_{i_{2}}^{*,(2)}(\lambda)$ are the spectral densities of the unobserved processes associated with levels *i*_1_ and *i*_2_ of factors A and B, respectively; and $f^{*}_{\varepsilon_{i_{1},i_{2},h}}(\lambda)$ is the spectral density of the idiosyncratic error component associated to individual *h* within such levels.

There is a relatively rich literature on Bayesian approaches for estimation of functions that can be used in the context of analyzing multiple signals. DiMatteo, Genovese, and Kass (2001) describe a Bayesian method for fitting curves to data drawn from a distribution in the exponential family. This approach assumes that the curves can be well approximated by splines with an unknown number of knots and unknown knot locations that are then inferred via reversible jump Markov chain Monte Carlo. The method is used to analyze individual (not multiple) time series data obtained from functional magnetic resonance imaging (fMRI) experiments. In such context the method of DiMatteo et al. (2001) is a time-domain approach that assumes that a given time series *y*(*t*) can be modeled as *y*(*t*)=*f*(*t*)+*ϵ*(*t*), with $f(t)=\sum_{j=1}^{k+2}\theta_{j} b_{j}(t)$, where *b*_*j*_(*t*) is the *j*th function of a cubic B-spline. In contrast, our proposed frequency-domain method aims to infer the spectral characteristics of multiple, not just a single, time series. A possible way of analyzing multiple times series in the spectral domain would consist on extending the method of DiMatteo et al. (2001) for fitting curves to the periodogram ordinates of the multiple time series recorded at various levels of the factors. Such extension would involve developing and implementing models that can handle multiple series given that the approach of DiMatteo et al. (2001) can only be directly applied to a single data set, e.g., the periodogram ordinates of an individual time series. Furthermore, the method of DiMatteo et al. (2001) applies to independent data (*y*_1_,*x*_1_),…,(*y*_*n*_,*x*_*n*_) that satisfy a model of the form *y*_*i*_|*x*_1_,…,*x*_*n*_∼*p*(*y*_*i*_|*f*(*x*_*i*_),*σ*). However, the periodogram ordinates are only asymptotically independent. Therefore, it would not be advisable to directly apply the free-knots splines method in order to estimate spectral densities. On the other hand, the method proposed here is based on the approaches of Choudhuri, Ghosal, and Roy (2004) and Macaro (2010), which provide consistent Bayesian estimates even if the periodogram ordinates are only asymptotically independent (see Choudhuri et al. 2004, pp. 1056–1057, and Macaro 2010, p. 384).

Regarding factorial temporal data, several authors have considered various time-domain and frequency-domain approaches. Some key references include, among others, Shumway (1970), Brillinger (1973, 1980), and Stoffer (1999). Shumway and Stoffer (2006) also summarize and illustrate several aspects of some of such approaches. As mentioned above, we follow Choudhuri et al. (2004) and Macaro (2010) to obtain Bayesian non-parametric posterior inference of the spectral representation of multiple time series, including the particular case of factorial spectral decompositions, by using Bernstein–Dirichlet prior distributions (Petrone 1999a, 1999b) on the spectral densities in (2). More specifically, Choudhuri et al. (2004) described a Bayesian approach to estimating the spectral density of a single stationary time series by imposing a non-parametric prior on such density through Bernstein polynomials. Then, Macaro (2010) proposed a mixture generalization of such approach in which each component in the spectral decomposition is identified using informative prior distributions. We extend the methods and algorithms of Macaro (2010) to provide a Bayesian non-parametric spectral analysis of multiple time series recorded during designed experiments that may involve several factors, various levels within each factor and several individuals. Bernstein–Dirichlet prior distributions have been successfully applied to analyze other types of data that arise in psychometric applications. For example, Karabatsos and Walker (2009) use a bivariate Bernstein–Dirichlet prior in the context of modeling test equating. Alternative Bayesian approaches to spectral density estimation of a single time series, including stationary and long-range dependence time series, as well as nonstationary time series with slowly varying dynamics or piecewise stationary time series, can be found in Carter and Kohn (1997), Gangopadhyay, Mallick, and Denison (1998), Liseo, Marinucci, and Petrella (2001), and Rosen, Stoffer, and Wood (2009), among others. A Bayesian approach for estimating the spectral density of multivariate stationary time series is presented in Rosen and Stoffer (2007).

The article is organized as follows. The details of the modeling approach, including some examples, are provided in Section 2. Section 3 summarizes the Markov chain Monte Carlo algorithm^1^ for posterior inference. In Section 4 we present the results of several simulation studies and discuss aspects of identification through the prior distributions. In Section 5 we analyze multiple time series of fMRI brain responses measured in individuals who participated in an experiment designed to study pain perception in humans. Finally, Section 6 presents the conclusions.

## Bayesian Spectral Decompositions of Factorial Time Series Data

### General Model Formulation

Let $\{y_{i_{1},\ldots,i_{D},h}(t)\}$ be a set of time series for *i*_*d*_=1:*N*_*d*_, $h=1:H_{i_{1},\ldots,i_{D}}$ and *t*=1:*T*. Assume that each time series $y_{i_{1},\ldots,i_{D},h}(t)$ can be decomposed as a sum of *D* unobservable components plus an idiosyncratic error term. That is,

3 $$ y_{i_1,\ldots,i_D,h}(t) = \sum_{d=1}^D \beta_{i_d}^{(d)}(t) + \varepsilon_{i_1,\ldots,i_D,h}(t), $$

where $\beta_{i_{j}}^{(j)}(t)$ and $\beta_{i_{k}}^{(k)}(t)$ are assumed to be independent for all *j*≠*k* and all *t*, and the $\varepsilon_{i_{1},\ldots,i_{D},h}(t)$ are assumed to be independent of the $\beta_{i_{j}}^{(d)}(t)$ processes for all *d* and also mutually independent. Furthermore, it is assumed that for a given *d*, $\beta_{i_{j}}^{(d)}(t)$ and $\beta_{i_{k}}^{(d)}(t)$ are independent for all *i*_*j*_≠*i*_*k*_ and all *t*, and that $\varepsilon_{i_{1},\ldots,i_{D},h}(t_{0})$ and $\varepsilon_{i_{1},\ldots,i_{D},h}(t_{1})$ are independent for all *t*_0_≠*t*_1_. This implies that the spectral density of $y_{i_{1},\ldots,i_{D},h}(t)$, denoted as $f_{y_{i_{1},\ldots,i_{D},h}}^{*}(\lambda)$, can be written as

4 $$ f_{y_{i_1,\ldots,i_D,h}}^*(\lambda) = \sum_{d=1}^D f_{i_d}^{*,(d)}(\lambda) + f_{\varepsilon_{i_1,\ldots,i_D,h}}^*(\lambda), $$

where $f_{i_{d}}^{*,(d)}(\lambda)$ and $f_{\varepsilon_{i_{1},\ldots,i_{D},h}}^{*}(\lambda)$ are, respectively, the spectral densities of the unobserved factors and the idiosyncratic error components. The spectral density is the Fourier transform of the theoretical autocovariance function. Under some regularity conditions it is a real and continuous function over *λ*∈(−*π*,*π*]. A further assumption requires the spectral densities to be bounded and bounded away from zero, ruling out long memory and band limited processes.

### Examples

#### Baseline Plus Single Factor Model

In this example we consider a one-way model represented as a baseline process plus one factor with two levels. Specifically, let *d*=1:2, *i*_1_=1, *i*_2_=1:2, *h*=1:2, and assume the following structure on $y_{1,i_{2},h}(t)$:

5 $$\begin{aligned} \mbox{Observations:}&\quad y_{1,i_2,h}(t) = \beta_{1}^{(1)}(t) + \beta_{i_2}^{(2)}(t) + \varepsilon_{1,i_2,h}(t),\quad \varepsilon_{1,i_2,h}(t) \sim N(0,1), \end{aligned}$$

6 $$\begin{aligned} \mbox{Baseline:}&\quad \beta_{1}^{(1)}(t) \sim \mathrm{AR} \bigl(\phi^{(1)}=0.9,1\bigr), \end{aligned}$$

7 $$\begin{aligned} \mbox{One factor, two levels:}&\quad \beta_{i_2}^{(2)}(t) \sim \left \{\begin{array}{l@{\quad }l} \mathrm{AR}(\phi_{1,1}^{(2)}=-0.8,\phi_{1,2}^{(2)}=-0.9,1) & i_2=1, \\ \mathrm{AR}(\phi_{2,1}^{(2)}=-1.5,\phi_{2,2}^{(2)}=-0.8,1) & i_2=2. \end{array} \right . \end{aligned}$$

Therefore, the baseline process $\beta_{1}^{(1)}(t)$ is an autoregressive process of order one, or AR(1), with coefficient 0.9, while the factor has two levels, the first level corresponds to an autoregressive process of order two, or AR(2), with two reciprocal characteristic roots each with modulus 0.949 and frequency 2.006 (period 3.132), and the second level is an AR(2) with two reciprocal characteristic roots each with modulus 0.894 and frequency 2.566 (period 2.449). The spectral representation is

8 $$ f_{y_{1,i_2,h}}^{*}(\lambda) = f_{1}^{*,(1)}( \lambda) +f_{i_2}^{*,(2)}(\lambda) + f_{\varepsilon_{i_1,i_2,h}}^*(\lambda), $$

with $f_{1}^{*,(1)}(\lambda)=1/[2\pi(1+(\phi^{(1)})^{2}-2\phi^{(1)} \cos(\lambda))]$, and

$$\begin{aligned} f_{i_2}^{*,(2)}(\lambda) =&1/\bigl[2\pi\bigl(1+\bigl( \phi_{i_2,1}^{(2)}\bigr)^2+2\phi_{i_2,2}^{(2)} +\bigl(\phi_{i_2,2}^{(2)}\bigr)^2+2\bigl( \phi_{i_2,1}^{(2)}\phi_{i_2,2}^{(2)} - \phi_{i_2,1}^{(2)}\bigr)\cos(\lambda) \\ &{}-4\phi_{i_2,2}^{(2)} \cos^{2}(\lambda)\bigr)\bigr]. \end{aligned}$$

**Figure 1.**
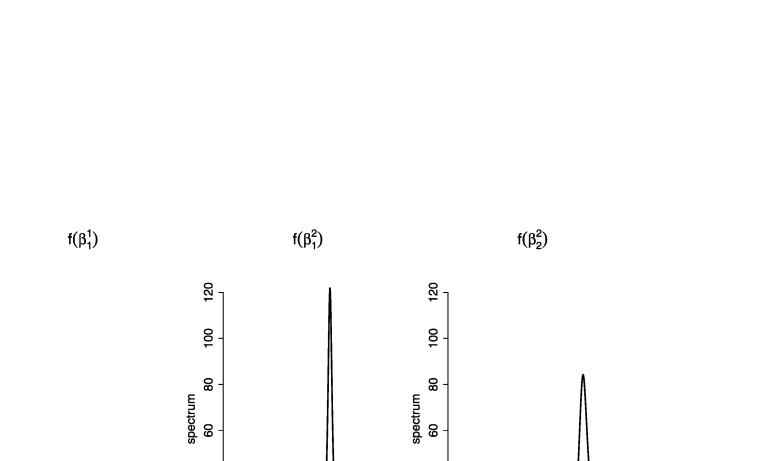
Spectral representation of the one-way factor model given in Equations (5)–(7).

Therefore, the decomposition in (8) is such that $f_{1}^{*,(1)}(\lambda)$ captures constant and persistent effects, while the $f_{i_{2}}^{*,(2)}(\lambda)$ spectra capture quasiperiodic dynamics that shift across the levels, as shown in Figure 1.

#### Two-Way Models

The probabilistic spectral decomposition in the example below corresponds to that of a two-way temporal model. In particular, assume that a two-way factor model with two levels in each factor is obtained by letting *d*=1:2, *i*_1_=1:2, *i*_2_=1:2, *h*=1:*H* and by assuming the following structure on $y_{i_{1},i_{2},h}(t)$:

9 $$\begin{aligned} \mbox{Observations:}\quad & y_{i_1,i_2,h}(t) = \beta_{i_1}^{(1)}(t) + \beta_{i_2}^{(2)}(t) + \varepsilon_{i_1,i_2,h}(t),\quad \varepsilon_{i_1,i_2,h}(t) \sim N(0,1), \end{aligned}$$

10 $$\begin{aligned} \mbox{First factor, two levels:}\quad & \beta_{i_1}^{(1)}(t) \sim \left \{\begin{array}{l@{\quad }l} N(0,1), & i_1=1, \\ \mathrm{AR}(\phi_{2,1}^{(1)}=1.46,\phi_{2,2}^{(1)}=-0.81,1), & i_1=2, \end{array} \right . \end{aligned}$$

11 $$\begin{aligned} \mbox{Second factor, two levels:}\quad & \beta_{i_2}^{(2)}(t) \sim \left \{\begin{array}{l} \mathrm{AR}(\phi_{1,1}^{(2)}=0.9,1),\\ \mathrm{AR}(\phi_{2,1}^{(2)}=-0.9,\phi_{2,2}^{(2)}=-0.81,1). \end{array} \right . \end{aligned}$$

**Figure 2.**
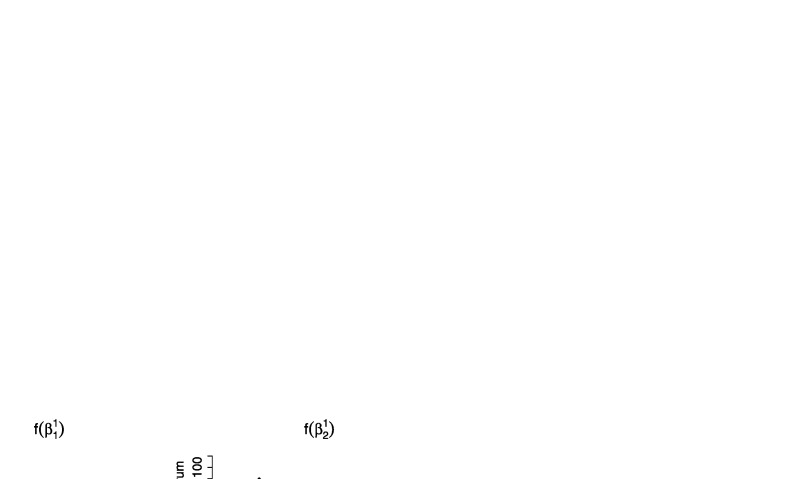
Spectral representation of the two-way factor model given in Equations (9)–(11).

Therefore, the spectral representation of $y_{i_{1},i_{2},h}(t)$ is given by

$$ f_{y_{i_1,i_2,h}}^*(\lambda)= f_{i_1}^{*,(1)}(\lambda) + f_{i_2}^{*,(2)}(\lambda) + f^*_{\varepsilon_{i_1,i_2,h}}(\lambda), $$

with $f_{1}^{*,(1)}(\lambda)$ the flat spectrum of a white-noise process and $f_{2}^{*,(1)}(\lambda)$, $f_{1}^{*,(2)}(\lambda)$ and $f_{2}^{*,(2)}(\lambda)$ the spectra of quasiperiodic AR(2) processes. The densities in the spectral decomposition of $y_{i_{1},i_{2},h}(t)$ are shown in Figure 2.

#### Interaction Effects

Oftentimes studies are characterized by the presence of one or more interaction terms. For example, a two-way time-domain model with interactions can be written as

12 $$ y_{i_1,i_2,h}(t) = \beta_{i_1}^{(1)}(t) + \beta_{i_2}^{(2)}(t) + \gamma_{i_1,i_2}^{(1,2)}(t) + \varepsilon_{i_1,i_2,h}(t), $$

where $\gamma_{i_{1},i_{2}}^{(1,2)}(t)$ are time series processes that represent possible interaction effects between factors 1 and 2 at the *i*_1_ and *i*_2_ levels. In general, if we have *D* factors, we can extend the model in (3) to allow for second order interactions as follows:

13 $$ y_{i_1,\ldots,i_D}(t)= \sum_{d=1}^D \beta_{i_d}^{(d)}(t) + \sum_{k=1}^D \sum_{l=1}^D \gamma_{i_k,i_l}^{(k,l)}(t) + \varepsilon_{i_1,i_2,h}(t). $$

The Bayesian non-parametric approach described in Section 3 is illustrated with one-way and two-way models with no interactions in Sections 4 and 5. However, the methodology can be applied to models that include interaction effects by adding such terms in Whittle’s likelihood approximation and by directly modeling the spectra of the interaction processes using the Bernstein–Dirichlet priors discussed in Section 2.4. Note that higher order interaction processes (e.g., third or higher order) can be added to (13) and modeled similarly.

As it will be shown in Section 4 with simulated data, when the number of factors increases, more restrictions need to be added to guarantee the identifiability of the different processes in the spectral decomposition. Therefore, instead of adding interaction effects and directly modeling the spectral densities of such processes, we propose simple post-processing calculations that can be used to explore if such interaction processes are present. We summarize such calculations in the case of a two-way model. Assume that $y_{i_{1},i_{2},h}(t)$ is represented as the two-way model process in (12). Such model can be written as $y_{i_{1},i_{2},h}(t) = \beta_{i_{1}}^{(1)}(t) + \beta_{i_{2}}^{(2)}(t)+\varepsilon^{\prime}_{i_{1},i_{2},h}(t)$, with $\varepsilon^{\prime}_{i_{1},i_{2},h}(t)= \gamma_{i_{1},i_{2}}(t)+ \varepsilon_{i_{1},i_{2},h}(t)$. If we were to analyze this scenario without implicitly considering the interaction terms, such terms would appear in the idiosyncratic factors $\varepsilon^{\prime}_{i_{1},i_{2},h}(t)$. Therefore, we could obtain estimates of the spectral densities of the interaction terms as follows:

1. Evaluate the common baseline across all the individuals (*i*_1_,*i*_2_,*h*). Compute
  $$f^{*}_{\bar{\varepsilon}}(\lambda)= \min_{i_1,i_2,h} f_{\varepsilon_{i_1,i_2,h}}^{*\prime}(\lambda).$$
2. Evaluate the corresponding individual residual. For each *λ* and each (*i*_1_,*i*_2_,*h*) compute
  $$f_{[\mathrm{residual}]_{i_1,i_2,h}}^{*\prime}(\lambda)=f_{\varepsilon_{i_1,i_2,h}}^{*\prime}(\lambda)-f^{*}_{\bar{\varepsilon}}(\lambda).$$
3. Evaluate the interaction term across all the individuals within the same group. For each *λ* and each (*i*_1_,*i*_2_) compute an estimate of the spectrum of the interaction process $\gamma_{i_{1},i_{2}}^{(1,2)}(t)$ as
  $$f^{*}_{\gamma_{i_1,i_2}}(\lambda)=\min_{h}f_{[\mathrm{residual}],i_1,i_2,h}^{*\prime}(\lambda). $$

By inspecting the posterior distribution of $f_{\gamma_{i_{1},i_{2}}}^{*}(\lambda)$ for each combination of (*i*_1_,*i*_2_), it can be determined if one or more interaction terms should be included in the analysis.

### Spectral Representation

Following a similar approach to that in Macaro (2010), we use a discrete version of Whittle’s approximation of the likelihood function (Whittle 1957, 1962) to estimate the factors. That is, the likelihood is approximated as

14 $$ L_T \bigl[I_{y_{i_1,\ldots,i_D,h}}( \lambda_{1:J})|f^{*,(1:D)}_{\beta}(\lambda_{1:J}),f^*_{\varepsilon}( \lambda_{1:J}) \bigr]= \prod_{j=1}^J \frac{\exp \{{-\frac{I_{y_{i_1,\ldots,i_D,h}}(\lambda_j)}{\sum_{d=1}^D f^{*,d}_{\beta_{i_d}}(\lambda_j) +f^*_{\varepsilon_{i_1,\ldots,i_D,h}}(\lambda_j)}} \}}{\sum_{d=1}^D f^{*,d}_{\beta_{i_d}}(\lambda_j) +f^*_{\varepsilon_{i_1,\ldots,i_D,h}}(\lambda_j)}, $$

where $I_{y_{i_{1},\ldots,i_{D},h}}(\lambda_{j})$ are the periodogram ordinates evaluated at the Fourier frequencies,

$$ \lambda_j=\frac{2\pi j}{T}\quad\mbox{for}\ j=0,1, \ldots,\lfloor{{T}/{2}}\rfloor:=J. $$

These summarize the information obtained from the data, while the $f_{\beta_{i_{d}}}^{*,d}(\lambda)$s are spectral ordinates to be estimated.

We now combine Whittle’s approximation to the likelihood in (14) with prior distributions on each $f_{\beta_{i_{d}}}^{*,d}(\lambda)$ to obtain posterior inference in the spectral domain. As discussed in Macaro (2010), prior elicitation is difficult since it involves the approximation of continuous spectral densities over the set of Fourier frequencies. Choudhuri et al. (2004) propose the use of Bernstein–Dirichlet prior distributions (Petrone 1999a, 1999b), while Macaro (2010) presents a mixture generalization of their work. Specifically, the spectrum of an observable time series is modeled as a weighted sum of several unobserved spectral densities in Macaro (2010). The resulting procedure is indeed more flexible, although the real novelty of the approach is the possibility of studying unobserved components from a non-parametric viewpoint. Here we extend Macaro (2010) to consider factorial designs with multiple time series. In other words, the models and methods described in this section lead to a non-parametric spectral representation of the unobserved components underlying multiple time series recorded in factorial experimental settings. Below we describe the prior structure and how posterior inference can be achieved under such prior structure. We also discuss some issues related to prior choice and summarize the steps of a Markov chain Monte Carlo (MCMC) algorithm for posterior inference.

### The Bernstein–Dirichlet Prior Distribution

The Bernstein polynomial prior developed by Petrone (1999a, 1999b) was used as a non-parametric prior to estimate densities on the [0,1] interval, and was then used by Choudhuri et al. (2004) for Bayesian estimation of spectral densities. This non-parametric prior hinges on the uniform convergence of

$$ \sum_{k=1}^KQ^{\dagger} \biggl( \frac{k-1}{K},\frac{k}{K} \biggr) \mathrm {Beta} _{(k,K-k+1)}(\lambda) $$

to *q*(*λ*), a continuous probability density on [0,1]. Here *Q* ^†^(*ϵ*_1_,*ϵ*_2_):=*Q*(*ϵ*_2_)−*Q*(*ϵ*_1_), with *Q*(*λ*) being the probability function associated to *q*(*λ*). In particular, the Bernstein–Dirichlet prior induces the weights *w*_1_,…,*w*_*K*_ of the mixture through a Dirichlet process, i.e.,

15 $$ q(\lambda)=\sum_{k=1}^Kw_{k} \mathrm {Beta} _{(k,K-k+1)}(\lambda), $$

with

16 $$ \varPi (w_{1},\ldots,w_{K}|K )=M \; \mathrm {Dirichlet} _{ (\theta_{1},\ldots,\theta_{K} )}(w_{1},\ldots,w_{K}), $$

where *θ*_1_,…,*θ*_*K*_ are the shape parameters, *M*>0 is the concentration parameter, and *K* determines how flexible is the prior, with larger values of *K* leading to more flexible distributions.

We now represent each of the spectral densities, $f_{\beta_{i_{d}}}^{*,(d)}(\lambda)$ in (4), through a Bernstein kernel. In order to implement Bernstein–Dirichlet priors in the spectral domain, such densities must be normalized to the interval [0,1]. Therefore, we can define a pseudo-spectral density function, $q_{y_{i_{1},\ldots,i_{D},h}}(\lambda)$, and a normalization parameter, $\tau_{y_{i_{1},\ldots,i_{D},h}}$, such that

$$ \tau_{y_{i_1,\ldots,i_D,h}}q_{y_{i_1,\ldots,i_D,h}}(\lambda):=\frac{\sigma^2_{y_{i_1,\ldots,i_D}}}{2\pi}q_{y_{i_1,\ldots,i_D,h}}( \lambda)=f_{y_{i_1,\ldots,i_D,h}}(\lambda), $$

where

$$\begin{aligned} \sigma_{y_{i_1,\ldots,i_D,h}}^2 =2\int_{0}^{\pi}f_{y_{i_1,\ldots,i_D,h}}^{*}(u)\,du = 2\int_{0}^{1}f_{y_{i_1,\ldots,i_D,h}}^{*}( \pi u)\,du :=2\pi\int_{0}^{1}f_{y_{i_1,\ldots,i_D,h}}( u)\,du. \end{aligned}$$

Then, the normalization parameters for the spectral representation of the unobserved factors $\beta_{i_{d}}^{(d)}(t)$ and the idiosyncratic errors $\varepsilon_{i_{1},\ldots,i_{D},h}(t)$ can be derived as follows:

$$\begin{aligned} q_{y_{i_1,\ldots,i_D,h}}(\lambda) =&\sum _{d=1}^D q_{\beta_{i_d}}^{(d)}(\lambda)+q_{\varepsilon_{i_1,\ldots,i_D,h}}(\lambda) \\ =&\sum _{d=1}^D \frac{f_{\beta_{i_d}}^{(d)}(\lambda)}{\tau_{\beta_{i_d}^{(d)}}}+ \frac{f_{\varepsilon_{i_1,\ldots,i_D,h}}(\lambda)}{\tau_{\varepsilon_{i_1,\ldots,i_D,h}}}, \end{aligned}$$

such that $\tau_{y_{i_{1},\ldots,i_{D},h}}=\sum_{d=1}^{D} \tau_{\beta_{i_{d}}^{(d)}} + \tau_{\varepsilon_{i_{1},\ldots,i_{D},h}}$, with

$$ {\tau}_{\beta_{i_d}^{(d)}}=\frac{{\sigma}^2_{{\beta_{i_d}^{(d)}}}}{2\pi} \quad\mathrm{and}\quad {\tau}_{\varepsilon_{i_1,\ldots,i_D,h}}=\frac{{\sigma}^2_{{\varepsilon_{i_1,\ldots,i_D,h}}}}{2\pi}. $$

Since ${\tau}_{\beta_{i_{1}}^{(d)}},\ldots,{\tau}_{\beta_{i_{D}}^{(d)}}$ and ${\tau}_{\varepsilon_{i_{1},\ldots,i_{D},h}}$ are proportional to the variances of the factors, inverse-gamma distributions can be used as priors. That is,

17 $$ \varPi[\tau_{(\cdot)}|a_{(\cdot)},b_{(\cdot)}]= \frac{b_{(\cdot)}^{a_{(\cdot)}}}{\varGamma [a_{(\cdot)} ]} \biggl[\frac{1}{\tau_{(.)}} \biggr]^{a_{(\cdot)}+1}\exp \biggl(- \frac{b_{(\cdot)}}{\tau_{(\cdot)}} \biggr). $$

The pseudo-spectral densities ${q}_{\beta_{i_{d}}}^{(d)}(\lambda)$ and ${q}_{\varepsilon_{i_{1},\ldots,i_{D},h}}(\lambda)$ will be discretized over the set of Fourier frequencies and estimated using Bernstein–Dirichlet priors.

Note that infinitely many choices of $\beta_{i_{d}}^{(d)}$ and $\varepsilon_{i_{1},\ldots,i_{D}}(t)$ would lead to the same $y_{i_{1},\ldots,i_{D},h}(t)$. The same is true in the spectral domain. Therefore, identifiability conditions need to be imposed. One way of doing so is by adding ANOVA-type restrictions (see Section 4.2). Alternatively, following Macaro (2010), identifiability can be achieved through proper specification of informative Bernstein–Dirichlet prior as follows. For each $q_{\beta_{i_{d}}}^{(d)}(\lambda)$ we have the representation in (15) and (16) characterized by weights $w_{k_{d}}^{i_{d},d}$ for $k_{d}=1:K_{i_{d}}^{d}$ and concentration parameter $M_{i_{d}}^{d}$. Similarly, for each $q_{\varepsilon_{i_{1},\ldots,i_{D},h}}(\lambda)$ we have a Bernstein–Dirichlet prior characterized by mixture weights $w^{\varepsilon_{i_{1},\ldots,i_{D},h}}_{k}$ for $k=1:K_{i_{1},\ldots,i_{D},h}$ and concentration parameter $M_{i_{1},\ldots,i_{d},h}^{\varepsilon}$. The concentration parameters control the variance of the Dirichlet processes. In other words, as $M_{i_{d}}^{d} \rightarrow 0$ the variance of the corresponding Dirichlet process increases; and as $M_{i_{d}}^{d} \rightarrow \infty $ such variance goes to zero. Therefore, instead of letting $M_{i_{d}}^{d} \rightarrow \infty$, we choose informed priors ($M_{i_{d}}^{d}< \infty$) on the $f_{i_{d}}^{(d)}(\lambda)$s. In practice, if we use the Dirichlet process characterization proposed by Sethuraman (1994), a natural choice is to fix $M_{i_{d}}^{d}=1$ (Choudhuri et al. 2004), which is equivalent to saying that the prior for $v^{i_{d},d}_{k_{d},l}$ for *l*=1:*L* is flat (see Section 3 for a detailed description of such parameters).

Theoretically, we could also set a prior on $K_{i_{d}}^{d}$ as proposed by Choudhuri et al. (2004), however, we fix these parameters in order to improve the speed and the stability of the MCMC algorithm (see Section 3 for further discussion). This will be illustrated in the analyses of simulated and real data sets in Sections 4 and 5. One of the main reasons for choosing this approach is that in many practical scenarios we have information about the unobserved components in the decomposition of the series that we may want to incorporate into the model through the prior distribution. For instance, electroencephalographic signals typically display activity in four main frequency bands (see, e.g., Prado, 2010), which induces a natural way of decomposing each signal into four unobserved components, one per frequency band.

Finally, it is important to notice the difference between $K_{i_{d}}^{d}$ and $M_{i_{d}}^{d}$. The parameter $K_{i_{d}}^{d}$ is similar to the bandwidth parameter used when smoothing the periodogram: a smaller value of $K_{i_{d}}^{d}$ leads to a higher degree of smoothing. On the other hand, $M_{i_{d}}^{d}$ indicates the degree of trust that we have in our prior distribution.

## Posterior Inference

The posterior associated with the likelihood function (14) and with the priors described above is not available in closed form. Nevertheless, samples from this distribution can be obtained with a Metropolis–Hastings Markov chain Monte Carlo (MCMC) algorithm. The original construction of the Dirichlet process is not suitable for MCMC schemes. Sethuraman (1994) proposed an alternative construction which has been implemented by Gelfand and Kottas (2002) for a more flexible algorithm. Our sampling procedure is a generalization of the algorithm in Macaro (2010) which, in turn, is an adaptation of the work of Choudhuri et al. (2004) for the analysis of spectral densities. Particularly, the MCMC algorithm outlined in Section 3.2 is not built for the weight parameters $w^{i_{d},d}_{1},\ldots,w^{i_{d},d}_{K_{i_{d}}^{d}}$ and $w^{\varepsilon_{i_{1},\ldots,i_{D},h}}_{1},\ldots,w^{\varepsilon_{i_{1},\ldots,i_{D},h}}_{K_{i_{1},\ldots,i_{D},h}}$, but for the set of parameters $r^{i_{d},d}_{k_{d},1},\ldots,r^{i_{d},d}_{k_{d},L}$, $v^{i_{d},d}_{k_{d},1},\ldots,v^{i_{d},d}_{k_{d},L}$, $r^{\varepsilon_{i_{1},\ldots,i_{D},h}}_{k_{h},1},\ldots,r^{\varepsilon_{i_{1},\ldots,i_{D},h}}_{k_{h},L}$, and $v^{\varepsilon_{i_{1},\ldots,i_{D},h}}_{k_{h},1},\ldots,v^{\varepsilon_{i_{1},\ldots,i_{D},h}}_{k_{h},L}$ such that

18 $$ w^{i_d,d}_{k_d}= \sum_{l=0}^L p^{i_d,d}_{k_d,l} \mathbf{1}_{ \{\frac{k_d-1}{K_{i_d}^d}<{r^{i_d,d}_{k_d,l}}\leq\frac{k_d}{K_{i_d}^d} \}}, $$

with

19 $$ p^{i_d,d}_{k_d,1}=v^{i_d,d}_{k_d,1}, \qquad p^{i_d,d}_{k_d,l}=v^{i_d,d}_{k_d,l}\prod _{i=1}^{l-1}\bigl(1-{v}^{i_d,d}_{k_d,i} \bigr), \qquad p^{i_d,d}_{k_d,0}=1-\sum _{l=1}^{L}p^{i_d,d}_{k_d,l}, $$

and

20 $$ w^{\varepsilon_{i_1,\ldots,i_D,h}}_{k_h} = \sum_{l=0}^L p^{\varepsilon_{i_1,\ldots,i_D,h}}_{k_h,l} \mathbf{1}_{ \{\frac{k_h-1}{K_{i_1,\ldots,i_D,h}}<{r^{\varepsilon_{i_1,\ldots,i_D,h}}_{k_h,l}}\leq\frac{k_h}{K_{i_1,\ldots,i_D,h}} \}}, $$

with

21 $$ \begin{aligned} p^{\varepsilon_{i_1,\ldots,i_D,h}}_{k_h,1} &= v^{\varepsilon_{i_1,\ldots,i_D,h}}_{k_h,1},\qquad p^{\varepsilon_{i_1,\ldots,i_D,h}}_{k_h,l}=v^{\varepsilon_{i_1,\ldots, i_D,h}}_{k_h,l} \prod_{i=1}^{l-1}\bigl(1-{v}^{\varepsilon_{i_1,\ldots,i_D,h}}_{k_h,i} \bigr), \\ p^{\varepsilon_{i_1,\ldots,i_D,h}}_{k_d,0}&= 1-\sum_{l=1}^{L}p^{\varepsilon_{i_1,\ldots,i_D,h}}_{k_h,l}, \end{aligned} $$

where the truncation level of the Dirichlet process is *L*=max(20,*T* ^1/3^) (Choudhuri et al. 2004).

### Prior Choice

The prior probabilities can be chosen by performing the following steps:

1. Choose the number and types of factors and idiosyncratic components. This will depend on the data structure and the particular decomposition of interest (e.g., one-way vs. two-way factor structures). For example, in the data analysis presented in Section 5, for each time series we use a model with two factors, where each factor has two levels, and one idiosyncratic component which tries to capture the individual-specific features that are not described by any of the two factors.
2. Determine the *a priori* spectral properties that characterize each factor and transform this information into kernel parameters $\theta^{i_{d},d}_{1},\ldots,\theta^{i_{d},d}_{K_{i_{d}}^{d}}$ for the corresponding Dirichlet process. For the idiosyncratic components the parameters can be set all equal to one to represent the prior assumption that these components are white noise. The prior for the remaining parameters $r^{i_{d},d}_{k_{d},1},\ldots,r^{i_{d},d}_{k_{d},L}$, $v^{i_{d},d}_{k_{d},1},\ldots, v^{i_{d},d}_{k_{d},L}$, $r_{k_{h},1}^{\varepsilon_{i_{1},\ldots,i_{D},h}},\ldots, r_{k_{h},L}^{\varepsilon_{i_{1},\ldots,i_{D},h}}$, and $v_{k_{h},1}^{\varepsilon_{i_{1},\ldots,i_{D},h}},\ldots,v_{k_{h},L}^{\varepsilon_{i_{1},\ldots,i_{D},h}}$ of the Bernstein representation are chosen according to the guidelines provided by Choudhuri et al. (2004).
3. Determine the flexibility of the priors on the factors: the smaller the value of $K_{i_{d}}^{d}$ and $K_{i_{1},\ldots,i_{D},h}$, the smoother the corresponding spectral densities. Notice that this is particularly useful to separate the white-noise effects—which are characterized by flat and smooth spectral densities—from other effects. It is worth to remember that although $K_{i_{d}}$ and $K_{i_{1},\ldots,i_{D},h}$ control for the smoothness of the spectra, they do not control for the strength of the prior distributions (this is achieved by changing the values of $M_{i_{d}}^{d}$ and $M_{i_{1},\ldots,i_{D},h}$).
4. Choose the $M_{i_{d}}^{d}$ and $M_{i_{1},\ldots,i_{D},h}$ concentration parameters: large values reduce the variance of the corresponding Dirichlet processes. Note for example that by letting $M_{i_{d}}^{d} \rightarrow\infty$ the underlying components become fixed (for more details see Macaro, 2010).
5. Determine the prior distributions for the normalization parameters. These parameters are proportional to the variances, therefore inverse gammas seem to be appropriate:
  22 $$ \tau_{(.)}\sim \mathrm {InvGamma} \bigl\{ s^\mathrm{prior}_{(.)}, \tilde{\tau}_{(.)}\bigl[s^\mathrm{prior}_{(.)}-1\bigr] \bigr\} $$
  with $s^{\mathrm{prior}}_{(.)}$ and $\tilde{\tau}_{(.)}$ chosen using the fact that
  23 $$ \mathrm {E}[\tau_{(.)} ]=\tilde{\tau}_{(.)}\quad \mbox{and} \quad \mathop {\mathrm {var}}[\tau_{(.)} ]=\frac{\tilde{\tau}_{(.)}^2}{s_{(.)}^\mathrm{prior}-2}. $$

### MCMC Algorithm

The posterior distribution associated with the likelihood function (14) and with the prior distributions described above is not available in closed form. The following Metropolis–Hastings MCMC algorithm can be used to obtain samples from the posterior distribution.

1. For each factor indexed by *d*=1:*D*
  - For each level of by *i*_*d*_=1:*N*_*d*_ of the factor *d*
    i. **Update v**. For each *l*=1:*L*
      Propose a candidate value for $v_{k_{d},l}^{i_{d},d}$.
      Evaluate the new set of $p_{k_{d},1}^{i_{d},d},\ldots,p_{k_{d},L}^{i_{d},d}$ and the corresponding new set of mixture weights $w_{1}^{i_{d},d},\ldots,w^{i_{d},d}_{K_{i_{d}}^{d}}$.
      Evaluate the new spectral density $f^{*,(d)}_{i_{d}}(\lambda)$ of the corresponding factor component and the likelihood (14).
      Decide whether to accept the proposed value of $v_{k_{d},l}^{i_{d},d}$.
    ii. **Update r**. For each *l*=0:*L*
      Propose a candidate value for $r_{k_{d},l}^{i_{d},d}$.
      Evaluate the new set of mixture weights $w_{1}^{i_{d},d},\ldots,w^{i_{d},d}_{K_{i_{d}}^{d}}$.
      Evaluate the new spectral density $f^{*,(d)}_{i_{d}}(\lambda)$ of the corresponding factor component and the likelihood (14).
      Decide whether to accept the proposed value of $r_{k_{d},l}^{i_{d},d}$.
    iii. **Update** ***τ***. Propose a candidate value of $\tau_{\beta_{i_{d}}}^{(d)}$ and evaluate the corresponding spectral density and the likelihood (14).
2. Given the updates for the levels *i*_*d*_ of each process *d*, update the corresponding idiosyncratic component.
  - For the set of indices (*i*_1_,…,*i*_*D*_,*h*),
    i. **Update v** for the spectral density $f_{\varepsilon_{i_{1},\ldots,i_{D},h}}^{*}(\lambda)$ as described in 1(a)i.
    ii. **Update r** for the spectral density $f_{\varepsilon_{i_{1},\ldots,i_{D},h}}^{*}(\lambda)$ as described in 1(a)ii.
    iii. **Update** ***τ*** for the spectral density $f_{\varepsilon_{i_{1},\ldots,i_{D},h}}^{*}(\lambda)$ as described in 1(a)iii.

The above algorithm was implemented in R (R Development Core Team 2007) using the MCMC package written by Martin and Quinn (2005).^2^ The algorithm is run for *I* iterations with *I* as large as needed to obtain MCMC convergence. This algorithm is an extension of the MCMC scheme of Macaro (2010) that allows us to consider factorial time series data. From the computational point of view this is rather complex; and, so, the computation time required to do the analysis increases with the number of factors, the number of levels within each factor, and the number of time series within each combination of factors. The proposed candidate values in Steps 1(a)i–ii and 2(a)i–ii are sampled from uniform distributions and those in Steps 1(a)iii and 2(a)iii are sampled from inverse-Gamma distributions whose moments are functions of the old parameter values (see Macaro 2010 for further details). In addition, the proposed values are accepted or rejected according to Metropolis–Hastings schemes.

## Simulation Studies

### Baseline Plus Single Factor Model

A data set with a total of four time series of length *T*=500 was simulated from the one-way model described in Section 2.2.1. The priors are chosen to be Beta kernels. Specifically, we chose a low frequency band-pass kernel for the baseline, ${q}_{\beta_{1}^{(1)}}(\lambda) \sim \mathrm {Beta} (1,20)$, and an approximately periodic kernel for the two levels of the remaining factor ${q}_{\beta^{(2)}_{1}}(\lambda) \sim \mathrm {Beta} (50,20)$ and ${q}_{\beta^{(2)}_{2}}(\lambda) \sim \mathrm {Beta} (50,20)$. Finally, ${q}_{\varepsilon_{i_{1},i_{2},h}}(\lambda) \sim \mathrm {Beta} (1,1)$ implies a white-noise assumption for the error term. The parameters controlling the flexibility of the representation are set to $K_{i_{1}}^{1}=40$, $K_{i_{2}}^{2}=40$ and $K_{i_{1},i_{2},h}^{3}=1$. This prior choice empowers the belief that the main factors must not capture white-noise effects; and, so, their spectra are not expected to be flat a priori. In addition, the parameters which control for the variances of the Dirichlet distributions are set to $M_{i_{d}}^{d}=1$ and $M_{\epsilon_{\cdot}}=1$.

**Figure 3.**
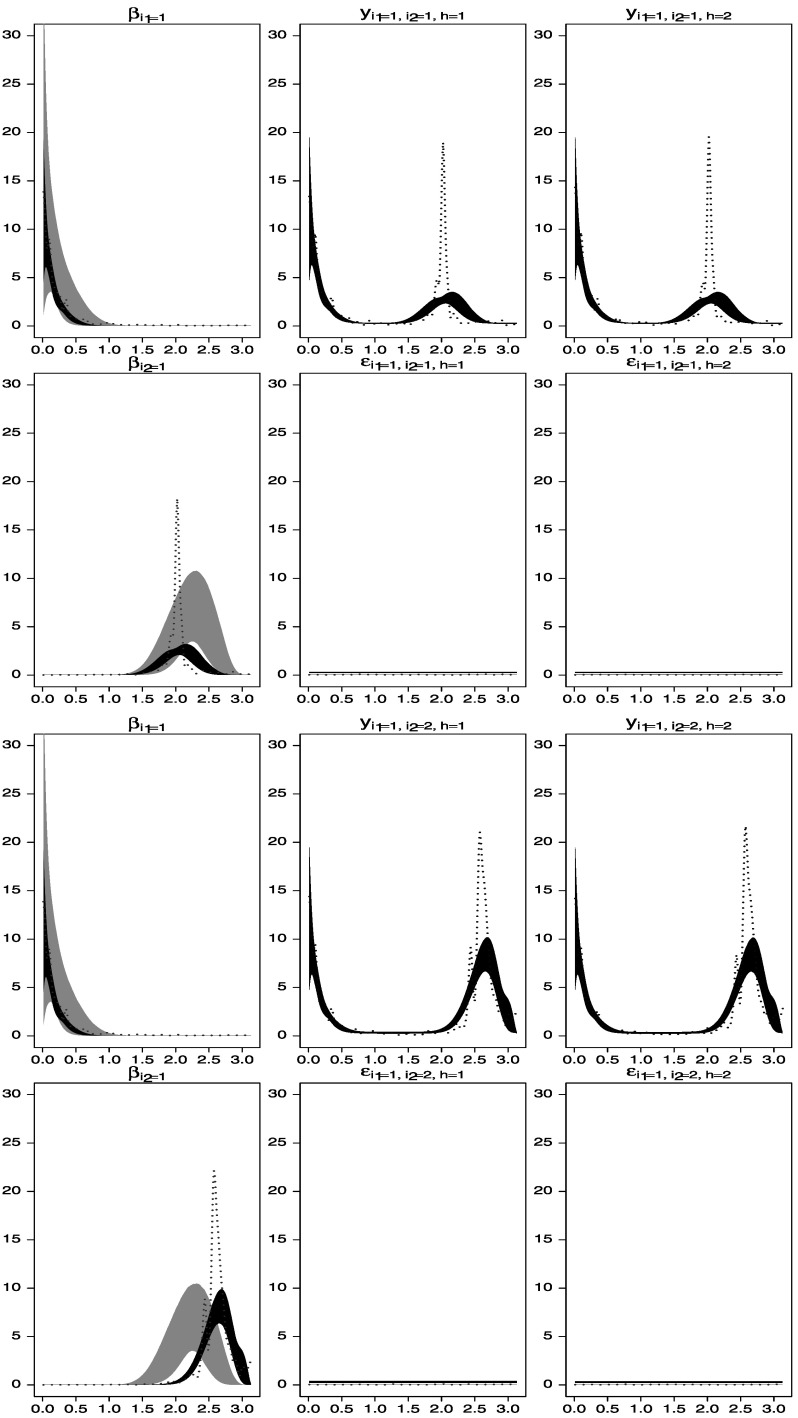
Spectral analysis of time series data simulated from the one-way model described in Section 2.2.1. *Gray areas* are central 95 % spectral prior distributions, while black areas are central 95 % spectral posterior distributions. The *dotted lines* represent estimators of the spectral densities obtained by smoothing the periodograms of the data. *First row*: estimated spectral densities of $\beta_{1}^{(1)}(t)$, *y*_1,1,1_(*t*), and *y*_1,1,2_(*t*). *Second row*: estimated spectral densities of $\beta_{1}^{(2)}(t)$, *ε*_1,1,1_(*t*), and *ε*_1,1,2_(*t*). *Third row*: estimated spectral densities of $\beta_{1}^{(1)}(t)$, *y*_1,2,1_(*t*), and *y*_1,2,2_(*t*). *Fourth row*: estimated spectral densities of $\beta_{2}^{(2)}(t)$, *ε*_1,2,1_(*t*) and *ε*_1,2,2_(*t*).

Figure 3 shows the prior and posterior distributions (gray and black areas, respectively), as well as estimators based on the smoothed periodograms for the spectral densities of the observed time series and the unobserved processes $\beta_{1}^{(1)}(t), \beta_{1}^{(2)}(t)$, and $\beta_{2}^{(2)}(t)$. Note that, in spite of the fact that the priors on the spectral densities of $\beta_{1}^{(2)}(t)$ and $\beta_{2}^{(2)}(t)$ had modes away from the correct periods, the estimated posterior spectral densities adequately capture the frequencies around 2.0 and 2.6.

### Two-Way Models

We analyze time series data simulated from the two-way model described in Section 2.2.2 with *d*=2, *i*_1_=1:2, *i*_2_=1:2, *h*=1:2, and *T*=500. We consider two alternative ways of selecting the prior distributions in this example. First we assume that the spectral density of the first level of the first factor, $\beta_{1}^{(1)}(t)$, as well as the spectral density of the *ε*_⋅,⋅,⋅_(*t*) processes are constant over all the frequencies. This is consistent with the assumption that these processes are white-noise processes. In addition, we assume weak and flat priors on the spectral densities of the remaining time series processes. Specifically, we have ${q}_{\beta_{2}^{(1)}}(\lambda) \sim \mathrm {Beta} (1.3,1.3)$, ${q}_{\beta^{(2)}_{1}}(\lambda) \sim \mathrm {Beta} (1.3,1.3)$, ${q}_{\beta^{(2)}_{2}}(\lambda) \sim \mathrm {Beta} (1.3,1.3)$. Notice that we purposely chose these kernels to be nearly flat to contrast the boundary bias (see Zhang & Karunamuni, 2010) which seems to affect weak and flat priors. The parameters controlling the flexibility of the representation are set again to $K_{i_{1}}^{1}=40$, $K_{i_{2}}^{2}=40$ and $K_{i_{1},i_{2},h}^{3}=1$. The parameters that control the variances of the Dirichlet distributions are set to $M_{i_{d}}^{d}=1$ and $M_{\epsilon_{\cdot}}=1$.

**Figure 4.**
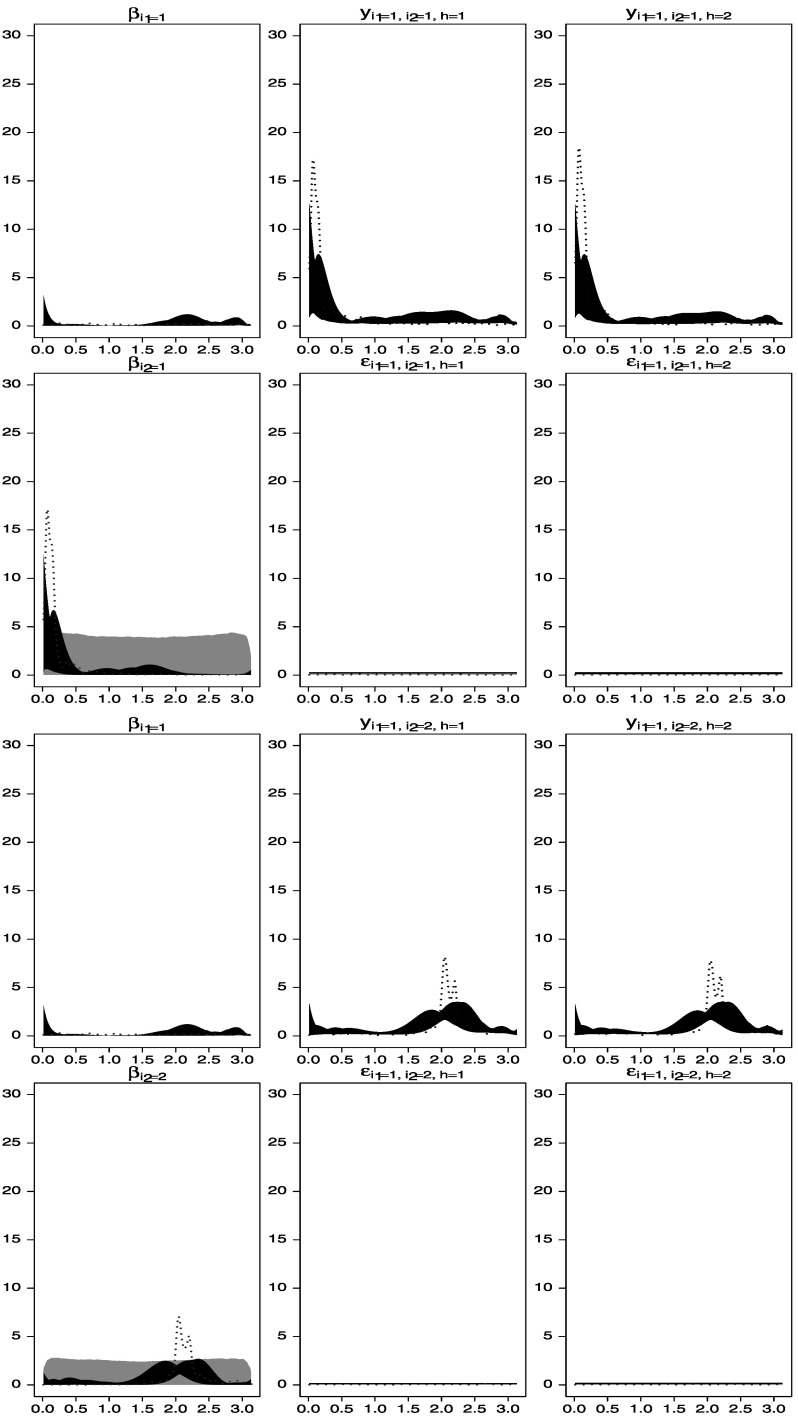
Spectral analysis of time series data simulated from the two-way model described in Section 2.2.2. *Gray areas* are central 95 % spectral prior distributions, while *black areas* are central 95 % spectral posterior distributions. The *dotted lines* represent estimators of the spectral densities obtained by smoothing the periodograms of the data. *First row*: estimated spectral densities of $\beta_{1}^{(1)}(t)$, *y*_1,1,1_(*t*), and *y*_1,1,2_(*t*). *Second row*: estimated spectral densities of $\beta_{1}^{(2)}(t)$, *ε*_1,1,1_(*t*), and *ε*_1,1,2_(*t*). *Third row*: estimated spectral densities of $\beta_{1}^{(1)}(t)$, *y*_1,2,1_(*t*), and *y*_1,2,2_(*t*). *Fourth row*: estimated spectral densities of $\beta_{2}^{(2)}(t)$, *ε*_1,2,1_(*t*) and *ε*_1,2,2_(*t*).

**Figure 5.**
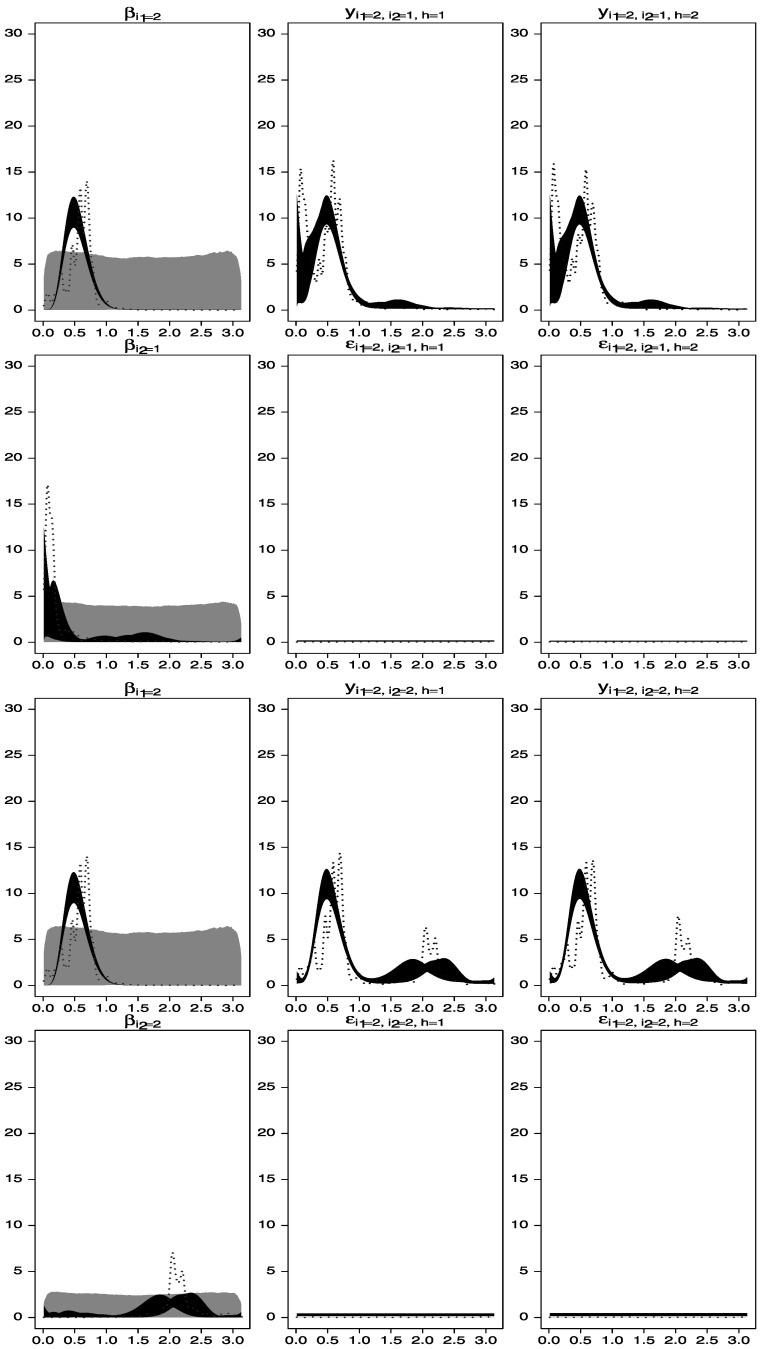
Spectral analysis of time series data simulated from the two-way model described in Section 2.2.2. *Gray areas* represent central 95 % spectral prior distributions, while *black areas* are central 95 % spectral posterior distributions. The *dotted lines* represent estimators of the spectral densities obtained by smoothing the periodograms of the data. *First row*: estimated spectral densities of $\beta_{2}^{(1)}(t)$, *y*_2,1,1_(*t*), and *y*_2,1,2_(*t*). *Second row*: estimated spectral densities of $\beta_{1}^{(2)}(t)$, *ε*_2,1,1_(*t*), and *ε*_2,1,2_(*t*). *Third row*: estimated spectral densities of $\beta_{2}^{(1)}(t)$, *y*_2,2,1_(*t*), and *y*_2,2,2_(*t*). *Fourth row*: estimated spectral densities of $\beta_{2}^{(2)}(t)$, *ε*_2,2,1_(*t*) and *ε*_2,2,2_(*t*).

By fixing the spectral densities of one of the levels of one of the factors and the spectral densities of the error term processes, identification of the remaining components is achieved, as shown in Figures 4 and 5.

**Figure 6.**
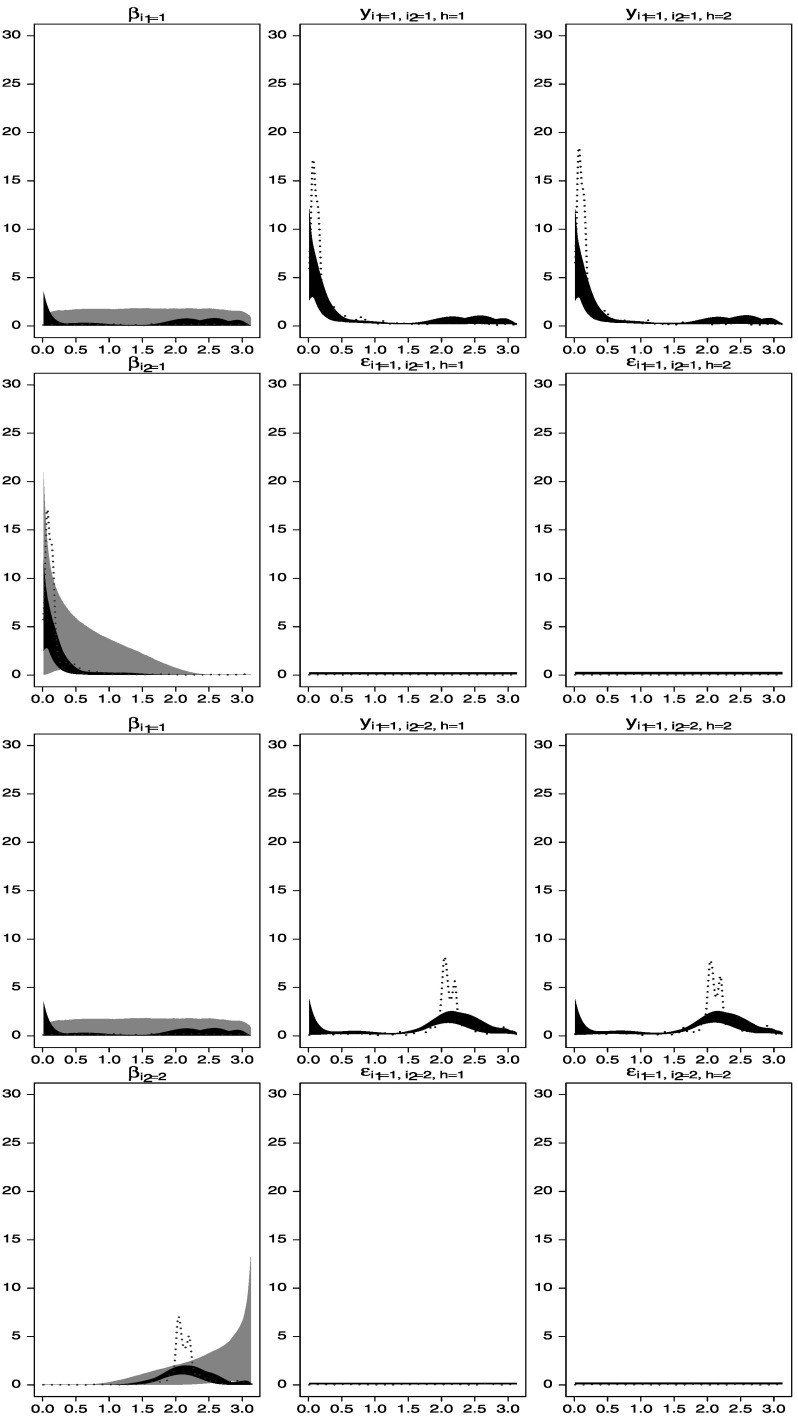
Spectral analysis of time series data simulated from the two-way model described in Section 2.2.2. *Gray areas* represent central 95 % spectral prior distributions, while *black areas* are central 95 % spectral posterior distributions. The *dotted lines* represent estimators of the spectral densities obtained by smoothing the periodograms of the data. *First row*: estimated spectral densities of $\beta_{1}^{(1)}(t)$, *y*_1,1,1_(*t*), and *y*_1,1,2_(*t*). *Second row*: estimated spectral densities of $\beta_{1}^{(2)}(t)$, *ε*_1,1,1_(*t*), and *ε*_1,1,2_(*t*). *Third row*: estimated spectral densities of $\beta_{1}^{(1)}(t)$, *y*_1,2,1_(*t*), and *y*_1,2,2_(*t*). *Fourth row*: estimated spectral densities of $\beta_{2}^{(2)}(t)$, *ε*_1,2,1_(*t*) and *ε*_1,2,2_(*t*).

**Figure 7.**
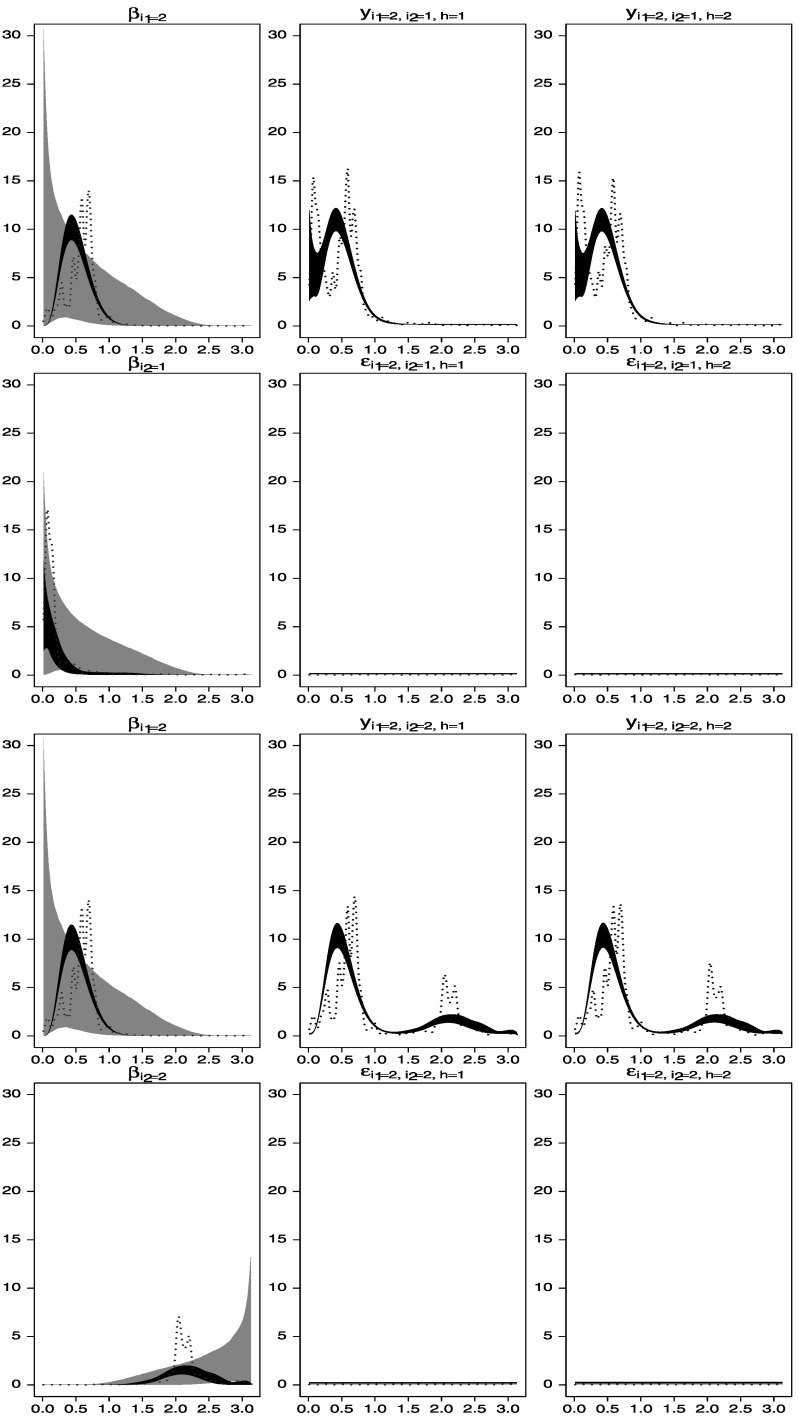
Spectral analysis of time series data simulated from the two-way model described in Section 2.2.2. *Gray areas* represent central 95 % spectral prior distributions, while *black areas* are central 95 % spectral posterior distributions. The *dotted lines* represent estimators of the spectral densities obtained by smoothing the periodograms of the data. *First row*: estimated spectral densities of $\beta_{2}^{(1)}(t)$, *y*_2,1,1_(*t*), and *y*_2,1,2_(*t*). *Second row*: estimated spectral densities of $\beta_{1}^{(2)}(t)$, *ε*_2,1,1_(*t*), and *ε*_2,1,2_(*t*). *Third row*: estimated spectral densities of $\beta_{2}^{(1)}(t)$, *y*_2,2,1_(*t*), and *y*_2,2,2_(*t*). *Fourth row*: estimated spectral densities of $\beta_{2}^{(2)}(t)$, *ε*_2,2,1_(*t*) and *ε*_2,2,2_(*t*).

The second scenario does not fix the spectra of any of the factor processes. It is assumed that the error processes have white-noise spectra a priori, and slightly more informative priors on the factor processes are considered. In particular we assume band-pass filter types of priors that emphasize smaller frequencies for $\beta_{1}^{(1)}(t)$ and $\beta_{2}^{(1)}(t)$ and larger frequencies for $\beta_{1}^{(2)}(t)$ and $\beta_{2}^{(2)}(t)$. The graphs in Figures 6 and 7 show that the implementation of a model with these band-pass filter priors produces results similar to those obtained under the white-noise assumption on $\beta_{1}^{(1)}(t)$ considered above and adequately captures the data structure.

### Further Identification Issues in Two-Way Models

In order to illustrate some additional features related to prior elicitation and corresponding posterior inference, we consider a larger simulated data set. Specifically, a total of 12 time series were simulated from a two-way model with the following structure:

24 $$ y_{i_1,i_2,h}(t) = \alpha_{i_1}^{(1)}(t) + \alpha_{i_2}^{(2)}(t)+ \varepsilon_{i_1,i_2,h}(t), $$

for *t*=1:500, *i*_1_=1:2, *i*_2_=1:2, and *l*=1:3, where

25 $$\begin{aligned} \alpha_1^{(1)}(t) \sim & \mathrm{AR} \bigl[2, (0.95,9),0.5\bigr], \qquad \alpha_2^{(1)}(t) \sim \alpha_1^{(1)}(t) + \mathrm{AR} \bigl[2, (0.95,3),0.5\bigr], \end{aligned}$$

26 $$\begin{aligned} \alpha_1^{(2)}(t) \sim & \mathrm{AR}[1, 0.86, 0.5] + \nu_1(t), \qquad \alpha_2^{(2)}(t) \sim \mathrm{AR}[1, 0.94, 0.5] + \nu_2(t), \end{aligned}$$

with $\varepsilon_{i_{1},i_{2},h}(t) \sim N(0,0.5)$ for all *i*_1_,*i*_2_ and *l*, *ν*_1_(*t*)∼*N*(0,0.5), and *ν*_2_(*t*)∼*N*(0,0.5). In the notation above, AR[2,(0.95,9),0.5] denotes a quasiperiodic autoregressive process of order two, or AR(2), with a pair of complex reciprocal roots with modulus 0.95, wavelength 9—or equivalently, frequency 0.698—and standard deviation 0.5. Similarly, AR[2,(0.95,3),0.5] denotes a quasiperiodic AR(2) process with a pair of complex reciprocal roots with modulus 0.95, wavelength 3—or frequency 2.094—and standard deviation 0.5; AR[1,0.86,0.5] denotes an AR(1) process with a reciprocal root of modulus 0.86 and standard deviation 0.5; and finally, AR[1,0.94,0.5] denotes an AR(1) with a reciprocal root of modulus 0.94 and standard deviation 0.5.

The structure above implies that the spectra of the series *y*_1,1,*h*_(*t*) and *y*_1,2,*h*_(*t*) will have a peak at the frequency *ω*_1_=0.698, in addition to the peak at zero related to the AR(1) components; while those of the series *y*_2,1,*h*_(*t*) and *y*_2,2,*h*_(*t*) will have an additional peak at the frequency *ω*_2_=2.094. We proceed to analyze these data with the Bayesian spectral non-parametric approach described in the previous sections. In particular, we assume the following underlying structure on the observed data:

$$ y_{i_1,i_2,h}(t) = \beta_{i_1}^{(1)}(t) + \beta_{i_2}^{(2)}(t) + \varepsilon_{i_1,i_2,h}(t). $$

Furthermore, we assume that, a priori, the spectra of $\beta_{i_{1}}^{(1)}(t)$ for *i*_1_=1:2 have a single peak around the frequency *ω*_1_=0.698, the spectra of $\beta_{i_{2}}^{(2)}(t)$ for *i*_2_=1:2 have only a peak at zero, and the spectra of $\varepsilon_{i_{1},i_{2},h}(t)$ are flat.

**Figure 8.**
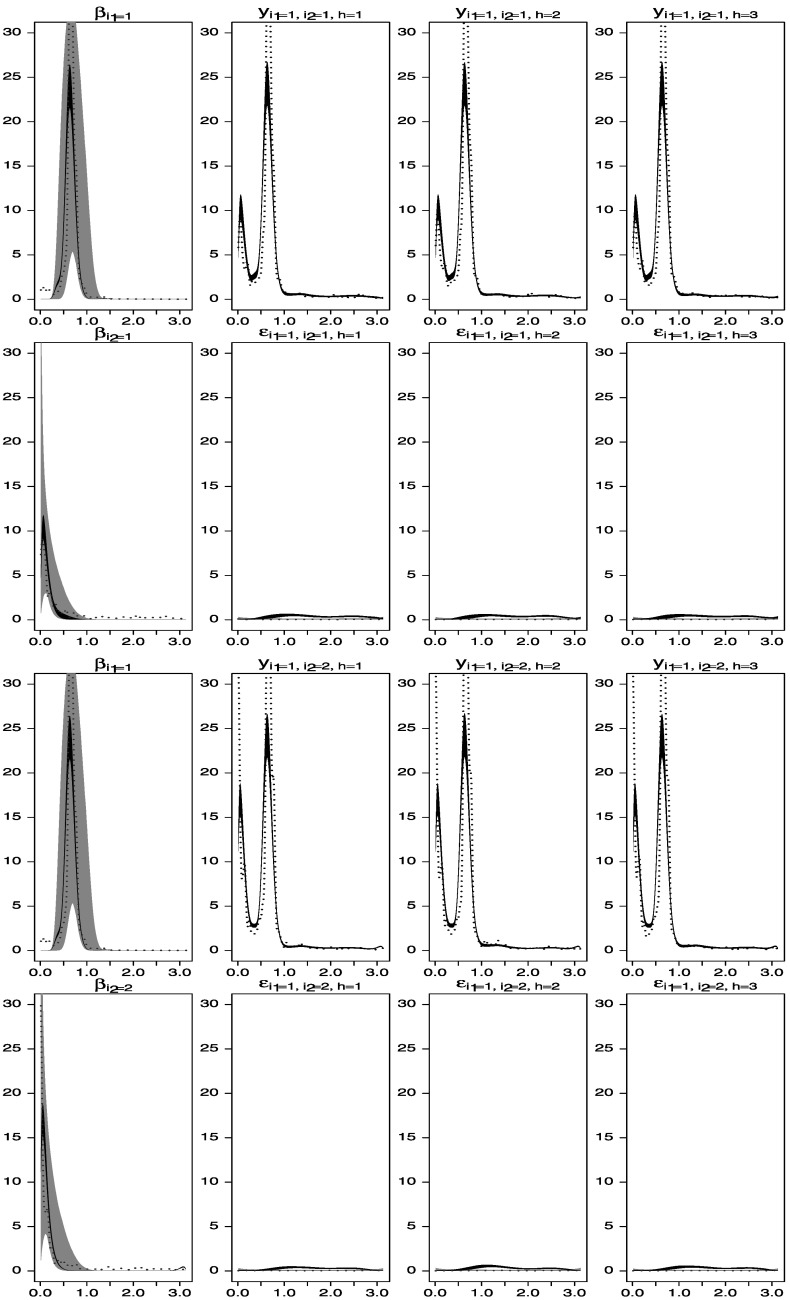
Spectral analysis of the time series data simulated from the model described in Section 4.3. *Gray areas* represent central 95 % of the prior distributions. *Black areas* represent central 95 % of the posterior distributions from the two-way model. The *dotted lines* represent periodogram-based estimators of the spectral densities of the individual series without considering the factor structure.

**Figure 9.**
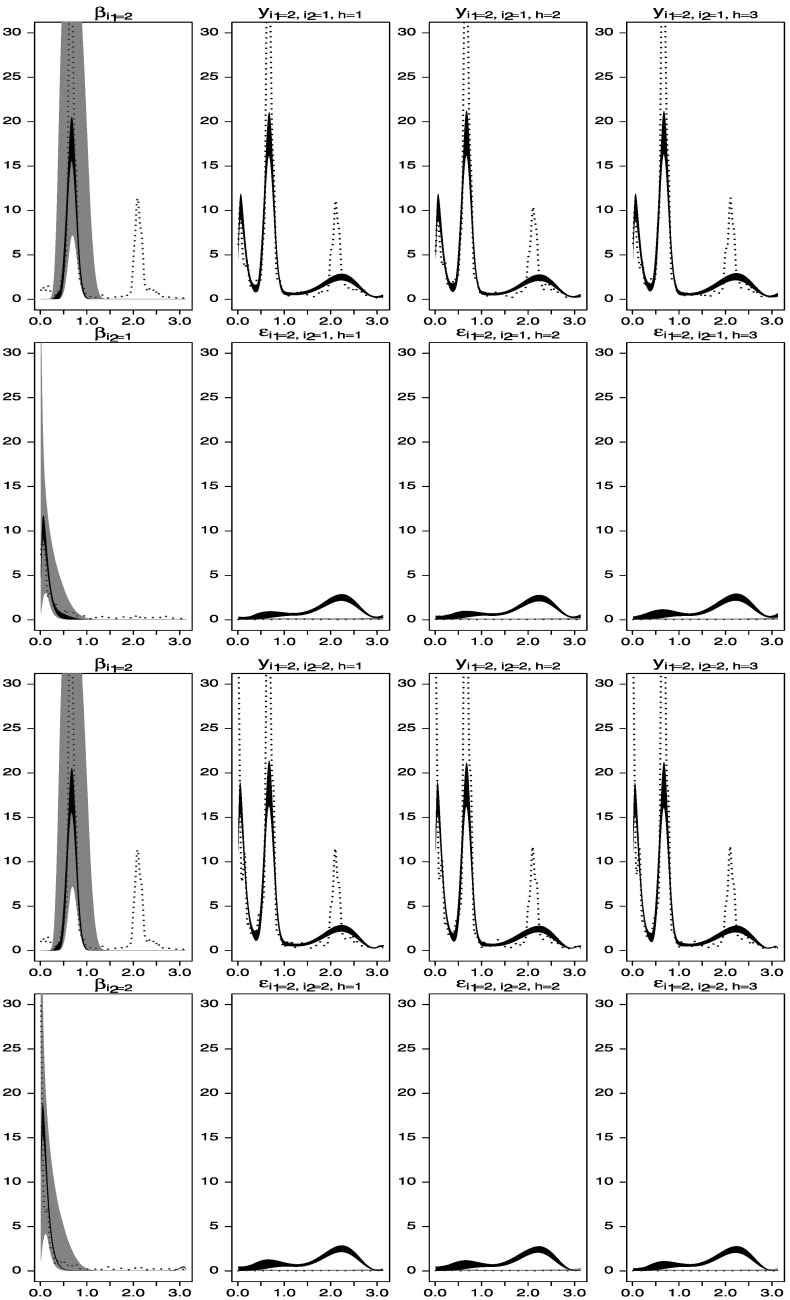
Spectral analysis of the time series data simulated from the model described in Section 4.3. *Gray areas* represent central 95 % of the prior distributions. *Black areas* represent central 95 % of the posterior distributions from the two-way model. The *dotted lines* represent periodogram-based estimators of the spectral densities of the individual series without considering the factor structure.

Figures 8 and 9 show the prior structure (gray areas). Note that this prior is closely describing the structure underlying the processes *y*_1,1,*h*_(*t*) and *y*_1,2,*h*_(*t*), but it misspecifies the structure of the processes underlying *y*_2,1,*h*_(*t*) and *y*_2,2,*h*_(*t*), completely missing the second peak at *ω*_2_=2.094.

Note that the error components, assumed to have flat spectra a priori under a white-noise structure, capture, a posteriori, whatever is left unexplained by the factor structure. Therefore, if an informative prior used on the spectra of a given factor misses certain frequency components present in the data, such components will be captured by the posterior distributions of the spectra of the idiosyncratic components, if relatively noninformative priors were given to these components. This is confirmed by the results shown in Figures 8 and 9. The prior distribution for $\beta_{2}^{(1)}(t)$ does not include one of the frequency components in the true $\alpha_{2}^{(1)}(t)$ and the posterior distribution of $\beta_{2}^{(1)}(t)$ does not capture the peak at *ω*_2_=2.094. Note, however, that this peak clearly appears in the estimated spectra of the six idiosyncratic error terms *ε*_2,1,*h*_(*t*) and *ε*_2,2,*h*_(*t*) for *h*=1:3, indicating that such structure is missing from the factorial decomposition of the process.

The analysis above that assumes informative priors on some of the unobserved components is performed in order to illustrate a couple of features about the spectral decomposition. First, the use of informative priors allow us to choose a particular decomposition of the series where some of the components may have a specific scientifically interpretable meaning a priori. For example, in this simulation study, we use a strong informative prior on the spectrum of $\beta_{2}^{(1)}(t)$ that identifies this time series process as one with activity in a particular frequency band. Alternatively, we could have used a more standard time-domain two-way representation of the data by assuming that the spectrum of one of the levels of one of the two factors, say the spectrum of $\beta_{1}^{(1)}(t)$, is flat (consistent with a white-noise prior assumption on this process). Such representation would also imply strong assumptions on the components that would not lead to the true unobserved representation in (24), (25), and (26), but that would allow us to adequately capture the structure of the spectra of the observed time series a posteriori. Researchers can therefore explore several spectral decompositions of the data by making different assumptions on the unobserved components, as long as such assumptions guarantee identifiability. The second model feature that we want to emphasize is that, regardless of the identifiability conditions used on the spectral characterization of the factors $\beta_{i_{d}}^{(d)}(t)$, the model will be able to adequately describe the structure of the data a posteriori if the priors on the spectra of $\varepsilon_{i_{1},i_{2},h}(t)$ are assumed to be flat and diffuse. As seen in the simulation study above, none of the priors on the spectra of $\beta_{i_{1}}^{(d)}(t)$ and $\varepsilon_{i_{1},i_{2},h}(t)$ had peaks on frequencies above 1.5, however, the estimated posterior spectra of *ε*_2,1,*h*_(*t*), *ε*_2,2,*h*_(*t*), and, therefore, the estimated posterior spectra of *y*_2,1,*h*_(*t*) and *y*_2,2,*h*_(*t*), show a peak at the *ω*_2_=2.094 frequency that does not appear in any of the spectra of the $y_{i_{1},i_{2},h}(t)$ series.

## Functional Magnetic Resonance Imaging Data

We consider the analysis of a data set from an experiment of Antognini, Buonocore, Disbrow, and Carstens (1998) in which functional magnetic resonance imaging (fMRI) was used to examine pain perception in humans. The data, previously analyzed in Stoffer (1999) and Shumway and Stoffer (2006), consist of consecutive measures of blood oxygenation level dependent (BOLD) signal intensities at nine locations in brain. The signals were measured in 26 awake and mildly anesthetized individuals who were presented with three types of periodic stimuli: brushing, heat, and shock. The stimuli were applied alternately for 32 seconds, with a sampling rate of one point every two seconds, and then stopped for 32 seconds.

Data were recorded by several sensors located on different parts of the brain, namely, Cortex 1, 2, 3 and 4, Caudate, Thalamus 1 and 2, and Cerebellum 1 and 2. Here we analyze only those related to the region Cortex 1. The 26 patients were divided into six groups depending on the type of stimulus they received (*Brush*, *Heat*, or *Shock*) and on whether they were awake or mildly anesthetized. Specifically, we have the following: (1) data from three patients in *Low* sedation state who were stimulated with a *Brush*; (2) data from five patients in *Low* sedation state stimulated by *Heat*; (3) data from four patients in *Low* sedation state stimulated by a *Shock*; (4) data from five *Awake* patients recorded while they were stimulated with a *Brush*; (5) data from four *Awake* patients stimulated by *Heat*; and finally, (6) data from five *Awake* patients stimulated by a *Shock*. Given this structure, a two-way model with one factor associated to the level of consciousness (*Low* and *Awake*) and another factor associated to the stimulus type (*Brush*, *Heat*, and *Shock*) was chosen to describe these data. Therefore, we consider a two-way model of the form

$$ y_{i_1,i_2,h}(t) = \beta_{i_1}^{(1)}(t) + \beta_{i_2}^{(2)}(t) + \varepsilon_{i_1,i_2,h}(t), $$

where $\beta_{i_{1}}^{(1)}(t)$ for *i*_1_=1:2 model the effects related to the consciousness level, $\beta_{i_{2}}^{(2)}(t)$ for *i*_2_=1:3 model the effects of the type of stimulus, and each $\varepsilon_{i_{1},i_{2},h}(t)$ is the idiosyncratic component modeling those effects specific to each patient that are not captured by the factor structure. Since the number of patients varies within each combination of consciousness level and stimulus type, we have *y*_1,1,*h*_(*t*) (*Low* and *Brush*) for *h*=1:3; *y*_1,2,*h*_(*t*) (*Low* and *Heat*) for *h*=1:5; *y*_1,3,*h*_(*t*) (*Low* and *Shock*) for *h*=1:4; *y*_2,1,*h*_(*t*) (*Awake* and *Brush*) for *h*=1:5; *y*_2,2,*h*_(*t*), (*Awake* and *Heat*) for *h*=1:4; and *y*_2,3,*h*_(*t*) (*Awake* and *Shock*) for *h*=1:5.

The priors are chosen to be Beta kernels. Specifically, we chose ${q}_{\beta_{1}^{(1)}}(\lambda) \sim \mathrm {Beta} (1.7,8.6)$; ${q}_{\beta_{2}^{(1)}}(\lambda)\sim \mathrm {Beta} (1.7,10.6)$; ${q}_{\beta^{(2)}_{1}}(\lambda) \sim \mathrm {Beta} (1.7,1.1)$; ${q}_{\beta^{(2)}_{2}}(\lambda) \sim \mathrm {Beta} (1.7,1.1)$; ${q}_{\beta^{(2)}_{3}}(\lambda) \sim \mathrm {Beta} (1.7,1.1)$; and ${q}_{\varepsilon_{i_{1},i_{2},h}}(\lambda) \sim \mathrm {Beta} (1,1)$ for all *i*_1_,*i*_2_ and *h*. The idea is that, a priori, the $\beta_{i_{1}}^{(1)}(t)$ processes aim to describe low frequency components, the $\beta_{i_{2}}^{(2)}(t)$ aim to describe high frequency components and $\varepsilon_{i_{1},i_{2},h}(t)$ are white-noise processes. For the remaining parameters, we set $K_{i_{1}}^{1}=30$ (cyclical), $K_{i_{2}}^{2}=100$ (long term) and $K_{i_{1},i_{2},h}^{3}=10$ (short-term/white noise) for *i*_1_=1:2, *i*_2_=1:3 and *h*=[(1:3),(1:5),(1:4),(1:5),(1:4),(1:5)]. The parameters that control the variances of the Dirichlet distributions were set to $M_{i_{d}}^{d}=1$ and $M_{\epsilon_{\cdot}}=1$.

**Figure 10.**
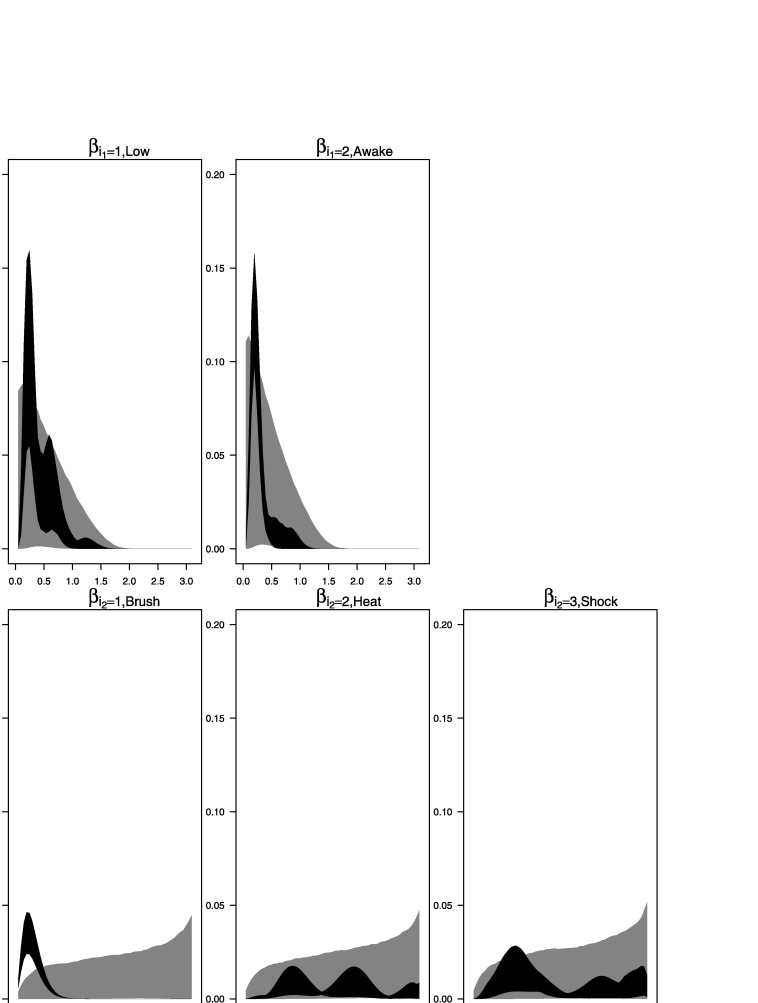
Spectral analysis of the fMRI data. The plots display the spectra of the factor processes. The *light gray areas* represent central 95 % of the prior distributions and the *dark gray areas* represent central 95 % of the posterior distributions.

**Figure 11.**
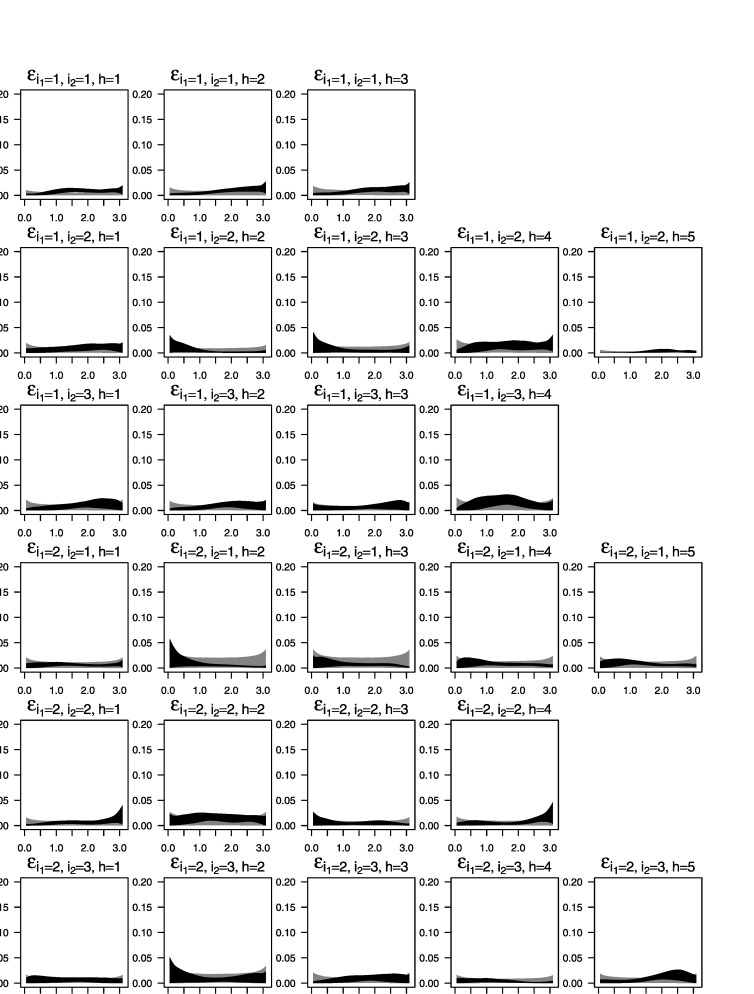
Spectral analysis of the fMRI data. The plots show the spectra of the idiosyncratic components for each of the 26 subjects. The *light gray areas* represent central 95 % of the prior distributions. The *dark gray areas* represent central 95 % of the posterior distributions.

Figure 10 shows the prior and posterior distributions (light and dark gray areas, respectively) of the spectra of the factors. Figure 11 shows the prior and posterior distributions (light and dark gray areas, respectively) of the spectra of the idiosyncratic processes for each of the 26 individuals.

From the graphs we can see that the posterior distributions of the spectral density for the process $\beta_{1}^{(1)}(t)$ related to *Low* sedation level and that for the process $\beta_{1}^{(2)}(t)$ related to *Awake* consciousness level show a marked peak around 0.19–0.22 which, given the sampling rate of the data, corresponds to the 64-second period band (or 1/64 cycles per second). The posterior spectral density of $\beta_{1}^{(1)}(t)$ (*Low*) shows more uncertainty in this frequency band than the posterior spectral density of $\beta_{2}^{(1)}(t)$ (*Awake*). In addition, the spectral density of $\beta_{1}^{(1)}(t)$ (*Low*) shows additional activity in higher frequency bands. Similarly, comparing the posterior estimates of the spectral densities of the processes $\beta_{1}^{(2)}(t)$, $\beta_{2}^{(2)}(t)$ and $\beta_{3}^{(2)}(t)$ associated, respectively, with the *Brush*, *Heat* and *Shock* stimuli, we see that the density for *Brush* shows a marked peaked around the 0.19–0.22 (again, the 64-second period band) that increases the power at this frequency in the estimated spectra of the subjects who received this type stimulus. For the other two types of stimuli, rather small peaks appear at higher frequencies. In summary, the posterior results based on the Bayesian non-parametric analysis for the data collected at the Cortex 1 location indicate that there is a significant difference in the power spectra at the 1/64 frequency band for patients that received the *Brush* stimulus.

## Conclusions

This work presents a Bayesian non-parametric framework for the analysis of multiple time series that are collected in factorial experimental designs. This approach is based on representing the prior distributions on the spectral densities with Bernstein–Dirichlet priors. It is an important extension of the univariate methods of Macaro (2010) in that it allows us to combine data and prior distributions on the unobserved spectral components in the decomposition of multiple time series. In particular, users can explore various decompositions of the observed time series processes and, consequently, various spectral decompositions, which can provide useful representations of the data.

Because of the possibility of extracting unobserved factors from a set of time series, this work is related to the well known literature on dynamic factor models (Geweke 1977; Sargent & Sims, 1977; Forni, Hallin, Lippi, & Reichlin, 2000; Stock & Watson, 2002, and many others). In our work we emphasize how the priors can be used to guarantee the identifiability of the different model components at the spectral level. Forni et al. (2000) and Stock and Watson (2002), on the other hand, derived their identification conditions from the properties of the principal component analysis.

We illustrate the main features and the performance of the Bayesian non-parametric approach in the analyses of simulated data and in functional magnetic resonance (fMRI) brain responses measured in awake and mildly anesthetized individuals who were presented with three types of stimuli. The posterior distributions on the specific spectral decomposition of the data that we chose—i.e., a decomposition in which each observed signal was represented as a sum of two unobserved components, one related to the level of consciousness and one to the stimulus, plus an idiosyncratic term—reveals differences in the power spectra at certain frequency bands for patients who received a particular stimulus. The spectral domain approaches presented here can be used to study data from a broad range of applications in which multiple time series are collected in the context of a designed experiment.

## Footnotes

1 The code is available upon request. Please email C. Macaro at christianmacaro@gmail.com.

2 The code is available upon request. Please email C. Macaro at christianmacaro@gmail.com.
